# Two-year long safety and efficacy of deferasirox film-coated tablets in patients with thalassemia or lower/intermediate risk MDS: phase 3 results from a subset of patients previously treated with deferasirox in the ECLIPSE study

**DOI:** 10.1186/s40164-020-00174-2

**Published:** 2020-08-10

**Authors:** Immacolata Tartaglione, Raffaella Origa, Antonis Kattamis, Michael Pfeilstöcker, Sibel Gunes, Susanne Crowe, Niamh Fagan, Beatrice Vincenzi, Giovan Battista Ruffo

**Affiliations:** 1 Department of Woman, Child and of General and Specialist Surgery, University of Campania Luigi Vanvitelli, Naples, Italy; 2 Ospedale Pediatrico Microcitemico “A.Cao,” A.O. “G.Brotzu”, Cagliari, Italy; 3 Division of Pediatric Hematology-Oncology, First Department of Pediatrics, National and Kapodistrian University of Athens, Athens, Greece; 4 Third Medical Department, Hanusch Hospital, Vienna, Austria; 5 Novartis Farma SPA, Origgio, Lombardy Italy; 6 Novartis Ireland Limited, Dublin, Ireland; 7 U.O.C. Ematologia e Talassemia, A.O. Civico-Di Cristina-Benfratelli, Piazza Nicola Leotta 4, 90127 Palermo, Italy

**Keywords:** Deferasirox, Chelation, Film-coated tablet, Iron overload, Long-term, Safety, Transfusion-dependent thalassemia, Myelodysplastic syndrome

## Abstract

**Background:**

Despite the proven benefits of iron chelation therapy (ICT) in the management of chronic iron overload and related complications, compliance to long-term ICT is challenging. Results from the ECLIPSE study, an open-label, randomized, multicenter, 2-arm, phase 2 study evaluated the safety of deferasirox dispersible tablet and film-coated tablet (FCT) formulations in patients with transfusion-dependent thalassemia (TDT) or very low, low, or intermediate risk myelodysplastic syndrome (MDS) treated over 24 weeks.

**Methods:**

The aim of the current study (a 2-year, open-label, multicenter, single-arm, phase 3 study) is to evaluate the long-term safety and efficacy of deferasirox FCT in a subset of patients with TDT or lower/intermediate-risk MDS treated for 2 years after the completion of 24 weeks of treatment with deferasirox in the ECLIPSE phase 2 study.

**Results:**

Of 53 patients enrolled, 34 (64.2%) completed treatment and study. Adverse events (AEs) reported in most patients (~ 70%) were of mild to moderate severity. Headache and diarrhea were the most frequently (> 25%) reported AEs. None of the serious AEs (including 1 death) were considered treatment related. No new safety signal was identified, and long-term safety of deferasirox FCT was consistent with the known safety profile of deferasirox. No major concerns associated with gastrointestinal tolerability, renal safety, or hematological abnormalities (thrombocytopenia/neutropenia) were reported during the 2 years. Patients receiving deferasirox FCT had a treatment compliance (by pill count) of ~ 90% and persistence (continuous use for ≥ 30 days) of > 95%. Reduction in serum ferritin level was almost consistent starting from week 2 across all post-baseline time points (relative reduction: month 6, 19%; month 12, 29%).

**Conclusions:**

The results from this 2-year interventional study suggest that the recommended dosing of deferasirox FCT, with better tolerability, palatability, and compliance, offers a favorable option of ICT for long-term management of iron overload and associated complications in TDT.

*Trial registration* ClinicalTrials.gov, NCT02720536. Registered 28 March 2016, https://www.clinicaltrials.gov/ct2/show/NCT02720536

## Introduction

Iron overload in chronic anemias represents a serious consequence of impaired hematopoiesis and repeated blood transfusions leading to end-organ damage, reduced quality of life, and decreased survival. Iron chelation therapy (ICT) can be a life-long requirement in chronic transfusion-dependent refractory anemias, including β-thalassemia, sickle-cell disease, and myelodysplastic syndrome (MDS) [1, 2]. Despite the proven benefit of ICT, patient compliance to long-term ICT is challenging [3]. Compliance with ICT is reported to influence the frequency and severity of iron overload–related complications [4–6], and a substantial increase in morbidity, mortality, and treatment cost has been seen among patients who are non-compliant to ICT [7].

Currently, 3 main iron chelators are available for clinical use: deferoxamine, deferiprone, and deferasirox. Parenterally administered deferoxamine was the mainstay of chelation therapy until the availability of oral chelators in 2005 [8]. Once-daily oral administration of deferasirox dispersible tablet (DT) formulation presented a better option, with greater compliance and quality of life over parenteral deferoxamine [9]. Currently, there are no direct comparison studies for the 2 oral chelators; however, once-daily simpler use of deferasirox has been projected to be a more cost-effective option than the thrice-daily administration of oral deferiprone in the management of long-term complications [7, 10]. The deferasirox film-coated tablet (FCT) formulation, with a simpler oral administration and improved palatability and gastrointestinal (GI) tolerability compared with deferasirox DT, offers a better option for optimal patient acceptance and improved compliance to long-term therapy [1, 11]. Thus, an appropriate choice of ICT plays an important role in patient compliance to chelation therapy.

The deferasirox FCT used in this study contained the same active substance of the iron chelator deferasirox and was strength-adjusted to achieve comparable exposure to the currently approved deferasirox DT. Deferasirox FCT is available in 3 dose strengths (90 mg, 180 mg, and 360 mg) and is dosed based on body weight. Unlike the DT, deferasirox FCT can be administered once daily orally without any need for dispersion, either on an empty stomach or with a light meal [11, 12].

Results from the ECLIPSE study, an open-label, randomized, multicenter, 2-arm, phase 2 study that evaluated the safety of deferasirox DT and FCT formulations in patients with transfusion-dependent thalassemia (TDT) or MDS (very low, low, or intermediate risk) treated over 24 weeks, have been published previously [11]. The purpose of the current study was to collect data on the long-term safety and efficacy of deferasirox FCT in a subset of patients with TDT or very low-, low-, or intermediate-risk MDS who had the possibility to continue treatment with deferasirox FCT after completion of 24 weeks of treatment in the ECLIPSE study. The study also aimed to collect efficacy data for deferasirox FCT in the reduction or maintenance of iron burden as measured by the serum ferritin level.

## Methods

### Key inclusion and exclusion criteria

Patients recruited at 14 European sites [Austria (n = 1), Greece (n = 3), Italy (n = 10)] who had completed the 24-week treatment in the ECLIPSE study with tolerance to deferasirox and fulfilled all eligibility criteria were included in this study. Patients were male or female aged ≥ 10 years with TDT or very low-, low- or intermediate-risk MDS who had received deferasirox DT at doses ≥ 30 mg/kg/day or ≥ 20 mg/kg/day, respectively as per clinical judgement. Patients had a transfusion history of ≥ 20 packed red blood cell units, were anticipated to be transfused with ≥ 8 units/year during the study, and had serum ferritin > 1000 ng/mL at screening. Key exclusion criteria in the study included patients with creatinine clearance (CrCl) below contraindication limit as per local label, serum creatinine (SCr) > 1.5 × upper limit of normal (ULN), alanine aminotransferase (ALT) > 5 × ULN (unless liver iron concentration was confirmed as > 10 mg Fe/dry weight within 6 months prior to screening), urine protein to creatinine ratio (UPCR) > 0.5 mg/mg, impaired GI function that may significantly alter the absorption of oral deferasirox, or clinical/laboratory evidence of active hepatitis B/hepatitis C infection.

### Study design

This was a 2-year, open-label, multicenter, single-arm, phase 3 study (NCT02720536) aimed to provide additional efficacy, safety, and drug exposure data following the ECLIPSE study [11].

Patients who were assigned to either the deferasirox DT or the deferasirox FCT arm and had completed the study period of 24 weeks with tolerance to deferasirox in the ECLIPSE study were allowed to participate in this study. It was planned that any patient continuing directly from the ECLIPSE study would receive the same dose of deferasirox FCT or an equivalent FCT dose at the start of this study corresponding to their DT dose at the end of the ECLIPSE study. As all patients had a lag period between the completion of the ECLIPSE study and the start of this study, i.e., patients who completed the ECLIPSE study switched to commercially available deferasirox DT or another ICT, they entered the current study following a washout period, with a starting dose that was based on clinical judgment. For each patient, the daily dose was calculated based on the patient’s actual body weight and then rounded up or down to the nearest whole tablet according to available strengths of deferasirox FCT tablets (90 mg, 180 mg, and 360 mg). Dose adjustments were allowed every 3 months based on serum ferritin levels and clinical judgment, with ± 3.5 to 7 mg/kg/day, up to a maximum dose of 28 mg/kg/day.

The study was conducted in accordance with the Good Clinical Practice guidelines and the Declaration of Helsinki and was approved by independent ethics committees at participating sites. The study is registered at https://www.clinicaltrials.gov/ct2/show/NCT02720536. Patients (or parents/guardians) provided written informed consent (or assent) prior to enrollment.

### Outcomes

The primary objective of the study was to evaluate the overall safety of the deferasirox FCT formulation, measured by the frequency and severity of adverse events (AEs) and changes in laboratory values of interest, such as SCr and CrCl. The secondary objective was to evaluate the efficacy of deferasirox FCT with respect to serum ferritin levels (decreased or maintained, according to individual therapeutic goal), measured by the absolute and relative changes in serum ferritin levels over time.

### Statistical evaluations

No formal sample size was calculated. A maximum of 58 patients (aged ≥ 10 years) were originally planned to be enrolled from the ECLIPSE study; 53 patients were enrolled into this study. There were no screening failures.

Data from all centers participating in this study were collected using electronic case record forms and pooled for analyses. The statistical software SAS^®^ (version 9.4) was used for analysis. The analyses were descriptive in nature; no hypothesis was tested. Data were summarized with respect to demographic and baseline characteristics, primary and secondary assessments, along with safety observations. All analyses were based on data collected as per protocol-scheduled assessments according to or including clinical judgment of the investigator.

Patient compliance to deferasirox FCT was evaluated using the count of deferasirox FCT by the relative consumed tablet count (%). Descriptive statistics, including 95% confidence intervals (CIs) for the mean relative consumed tablet counts, were provided. Persistence, defined as continuous use of deferasirox FCT without a gap for ≥ 30 or ≥ 60 days over a fixed time interval of interest, was summarized at month 3, month 6, month 9, and month 12. The incidence of any treatment-emergent AEs (i.e., AEs from the start of study treatment to 30 days after the last intake of study drug), overall and by maximum grade severity (mild, moderate, or severe as reported by the clinician), were summarized using frequency counts and percentages of patients. For each of the laboratory parameters of interest (SCr, CrCl, ALT, and aspartate aminotransferase [AST]), the worst post-baseline values were summarized as shift tables based on notable/extended ranges. For serum ferritin and hematological parameters (red blood cells [RBCs], platelets, total white blood cells, hemoglobin, and hematocrit), the absolute mean and the relative mean changes from baseline were summarized at each post-baseline visit.

## Results

**Fig. 1:**
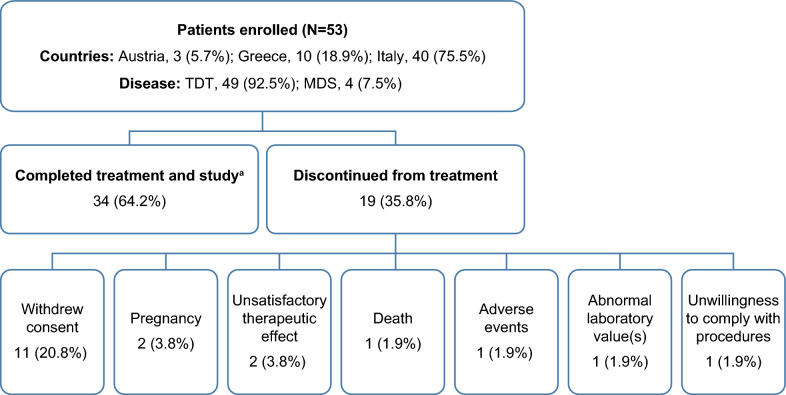
Patient disposition. *MDS* myelodysplastic syndrome, *TDT* transfusion-dependent thalassemia. ^a^The 34 patients who completed the study had received treatment for at least 24 months

Overall, 53 patients were enrolled from 3 countries across Europe, of whom, 19 (35.8%) discontinued early from treatment (Fig. 1).

Of the 53 patients enrolled, the majority (49 patients [92.5%] including 3 patients aged < 18 years) had TDT, and 4 patients (7.5%) had MDS (all aged ≥ 60 years). Most patients were Caucasians (94.3%), and females comprised 66.0% of the study population. All patients had a history of prior ICT. Demographics and other baseline characteristics are described in Table 1.

**Table 1:** Demographics and other baseline characteristics

Demographic variable	Deferasirox FCT, N = 53
Age (years), mean (SD)	32.9 (13.16)
Age category (years), n (%)	
< 18	3 (5.7)
≥ 18 to < 50	46 (86.8)
≥ 50 to < 65	1 (1.9)
≥ 65^a^	3 (5.7)
Sex, n (%)	
Male	18 (34.0)
Female	35 (66.0)
BMI (kg/m^2^), mean (SD)	23.1 (3.81)
Predominant race, n (%)	
Caucasian	50 (94.3)
Asian	1 (1.9)
Other^b^	2 (3.8)
Main underlying disease, n (%)	
MDS IPSS-R risk stratification	4 (7.5)
Very low risk	2 (3.8)
Low risk	1 (1.9)
Intermediate risk	1 (1.9)
TDT	49 (92.5)
Time since diagnosis (years), mean (SD)	27.1 (9.97)
MDS	5.1 (1.91)
TDT	28.9 (7.99)
Prior ICT (received during the ECLIPSE study), n (%)	
Deferasirox DT	27 (50.9)
Deferasirox FCT	26 (49.1)
Last ICT received before deferasirox FCT in the current study, n (%)	
Deferiprone	5 (9.4)
Deferoxamine and deferiprone	3 (5.7)
Deferasirox monotherapy	45 (84.9)
Transfusion history	
Total number of transfusions received over the past 12 months, mean (SD)	24.8 (10.52)
Time since last blood transfusion (days), mean (SD)	8.0 (10.21)
Baseline serum ferritin (µg/L), mean (SD)	2524 (1746)

*BMI* body mass index, *DT* dispersible tablet, *FCT* film-coated tablet, *ICT* iron chelation therapy, *IPSS-R* Revised International Prognostic Scoring System, *MDS* myelodysplastic syndrome, *SD* standard deviation, *TDT* transfusion-dependent thalassemia

^a^The oldest patient was 77 years old

^b^Other included two patients who self-identified as white

In most patients (84.9%), deferasirox monotherapy was the last ICT before study treatment. The majority of patients (71.7%) had received prior medication or therapy. The most common (> 15%) prior medications by the Anatomical Therapeutic Chemical classification system class included vitamin D and vitamin D analogues (21 patients [39.6%]), proton pump inhibitors (PPIs; 10 patients [18.9%]), sequential preparations of progestogens and estrogens (9 patients [17.0%]), fixed combinations of progestogens and estrogens (9 patients [17.0%]), and thyroid hormones (8 patients [15.1%]).

Almost all patients (52 [98.1%]) received concomitant medication or significant non-drug therapies on or after the start of study treatment. The most common (> 30%) concomitant medications by ATC class included vitamin D and vitamin D analogues (28 patients [52.8%]), anilides (23 patients [43.4%]), antibiotics (18 patients [34.0%]), other agents for local oral treatment (18 patients [34.0%]), PPIs (17 patients [32.1%), and other antibiotics for topical use (17 patients [32.1%]).

All patients received at least 3 transfusions during the study with a maximum of 91 transfusions received by 1 patient. The majority of patients (44 [83.0%]; including the 4 MDS patients) received 10 to < 60 transfusions (Additional file 1: Table S1).

### Exposure to treatment and compliance

The mean (standard deviation [SD]) duration of exposure to treatment was 571.5 (215.3) days, with most patients (50 [94.3%]) being treated for at least 20 weeks. The majority of patients receiving deferasirox FCT were in the longest exposure category (≥ 100 weeks, 45.3%). The sum of each patient’s treatment exposure to deferasirox FCT was 82.9 patient-years. Almost all patients (52 [98.1%]) received deferasirox FCT at an average dose of ≥ 10.5 mg/kg/day, with 21.3 (4.63) mg/kg/day as the mean (SD) average actual deferasirox FCT dose received (Table 2). MDS patients (n = 4) received an average dose of ≥ 15 mg/kg/day, with 15.6 (1.25) mg/kg/day as the mean (SD) average actual deferasirox FCT dose received.

**Table 2:** Exposure to treatment and compliance

	Deferasirox FCT, N = 53
Duration of exposure (days), mean (SD)	571.5 (215.28)
Duration of exposure categories (weeks), n (%)	
< 24	6 (11.3)
24 to < 52	4 (7.5)
52 to < 72	8 (15.1)
72 to < 100	11 (20.8)
≥ 100	24 (45.3)
Patient treatment years	82.9
Average actual dose (mg/kg/day), mean (SD)	21.3 (4.63)
Average actual dose category (mg/kg/day), n (%)	
< 10.5	1 (1.9)
10.5 to < 17.5	10 (18.9)
17.5 to < 24.5	27 (50.9)
≥ 24.5	15 (28.3)
Compliance	
Relative consumed tablet count (%), mean (SD)	90.2 (10.07)
Persistence	
Continuous use of deferasirox FCT with no interruption for ≥ 60 days, n (%)	
Up to 3 months (n = 53)	53 (100.0)
Up to 6 months (n = 51)	51 (100.0)
Up to 9 months (n = 47)	47 (100.0)
Up to 12 months (n = 46)	46 (100.0)
Continuous use of deferasirox FCT with no interruption for ≥ 30 days, n (%)	
Up to 3 months (n = 53)	52 (98.1)
Up to 6 months (n = 51)	50 (98.0)
Up to 9 months (n = 47)	46 (97.9)
Up to 12 months (n = 46)	44 (95.7)

F*CT* film-coated tablet, *SD* standard deviation

Patients had a mean relative consumed tablet count of 90.2% (95% CI 87.4–93.0). The proportions of patients with continuous use of deferasirox FCT with no interruption for ≥ 30 days and ≥ 60 days were 95.7% (n = 44) and 100.0% (n = 46), respectively, at 12 months.

### Efficacy

**Fig. 2:**
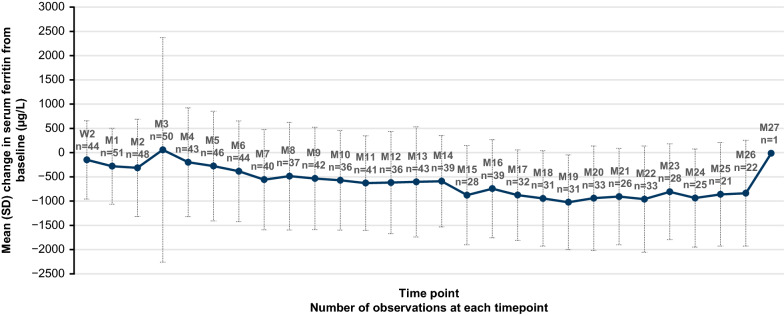
Change in serum ferritin from baseline (µg/L) by time point. *M* month, *SD* standard deviation, *W* week. Error bars represent the ± SD values for the respective mean values

A decrease in mean serum ferritin levels was observed from week 2 onward, except for month 3, though the median serum ferritin levels showed a consistent decrease across all post-baseline time points. The mean (SD) actual decrease in serum ferritin level was 385 (1039) µg/L from baseline to month 6, 618 (1054) µg/L from baseline to month 12, and 937 (1013) µg/L from baseline to month 24 (Fig. 2). The decrease (relative change in % [SD]) was higher at month 24 than at month 12 and month 6 (37% [47] vs 29% [33] vs 19% [33]). The mean (SD) actual decrease in serum ferritin level from baseline to month 27 was reported as 15 µg/L (relative change in percentage, 3%) in 1 patient at month 27.

### Safety

#### Adverse events

Of the 53 patients, almost all (52 [98.1%]) reported at least 1 AE regardless of study drug relationship. Additional file 1: Table S2 provides an overview of all AEs.

The most common AEs by preferred term (> 20%) were headache (26.4%), diarrhea (26.4%), pyrexia (24.5%), nausea (22.6%), vomiting (22.6%), cough (22.6%), and upper abdominal pain (20.8%). The most common AEs reported in > 50% of patients by system organ class (SOC) during the treatment with deferasirox FCT were related to infections and infestations (40 [75.5%]; influenza, rhinitis, gastroenteritis, pharyngitis, and urinary tract infection occurred in > 10% of patients by preferred term), followed by GI disorders (36 [67.9%]; diarrhea, nausea, vomiting, upper abdominal pain, and abdominal pain occurred in > 10% of patients by preferred term) (Table 3). None of the AEs in the infections and infestations SOC were treatment related. Of the AEs that were suspected to be treatment related in 20 patients (37.7%), increased UPCR (11.3%), diarrhea (9.4%), increased blood creatinine (7.5%), gastritis (5.7%), and proteinuria (5.7%) were the most common (reported in > 5% of patients) AEs (by preferred term). Of these suspected treatment-related AEs, increased blood creatinine and diarrhea (both of mild severity) were reported in 2 MDS patients.

**Table 3:** Common adverse events (>10%) reported by maximum grade of severity

AEs (N = 53) System organ class Preferred term	Overall, n (%)	Mild, n (%)	Moderate, n (%)	Severe, n (%)
Number of patients with at least one event	52 (98.1)	11 (20.8)	27 (50.9)	14 (26.4)
Infections and infestations	40 (75.5)	15 (28.3)	21 (39.6)	4 (7.5)
Rhinitis	8 (15.1)	8 (15.1)	0	0
Gastroenteritis	7 (13.2)	6 (11.3)	0	1 (1.9)
Pharyngitis	7 (13.2)	4 (7.5)	3 (5.7)	0
Urinary tract infection	6 (11.3)	3 (5.7)	3 (5.7)	0
Gastrointestinal disorders	36 (67.9)	24 (45.3)	12 (22.6)	0
Diarrhea	14 (26.4)	11 (20.8)	3 (5.7)	0
Nausea	12 (22.6)	9 (17.0)	3 (5.7)	0
Vomiting	12 (22.6)	9 (17.0)	3 (5.7)	0
Upper abdominal pain	11 (20.8)	7 (13.2)	4 (7.5)	0
Abdominal pain	10 (18.9)	7 (13.2)	3 (5.7)	0
General disorders and administration site conditions	24 (45.3)	16 (30.2)	8 (15.1)	0
Asthenia	10 (18.9)	8 (15.1)	2 (3.8)	0
Influenza	9 (17.0)	3 (5.7)	6 (11.3)	0
Pyrexia	13 (24.5)	7 (13.2)	6 (11.3)	0
Respiratory, thoracic and mediastinal disorders	20 (37.7)	13 (24.5)	7 (13.2)	0
Cough	12 (22.6)	9 (17.0)	3 (5.7)	0
Oropharyngeal pain	9 (17.0)	6 (11.3)	3 (5.7)	0
Investigations^a^	17 (32.1)	6 (11.3)	9 (17.0)	2 (3.8)
Urine protein/creatinine ratio increased	8 (15.1)	5 (9.4)	1 (1.9)	2 (3.8)
Musculoskeletal and connective tissue disorders	17 (32.1)	14 (26.4)	1 (1.9)	2 (3.8)
Musculoskeletal pain	6 (11.3)	6 (11.3)	0	0
Nervous system disorders	16 (30.2)	10 (18.9)	5 (9.4)	1 (1.9)
Headache	14 (26.4)	9 (17.0)	4 (7.5)	1 (1.9)

Proportion of patients with AEs > 10% reported by preferred term and grouped by system organ class

*AE* adverse event

^a^Abnormal laboratory values reported as AEs

The maximum grade of severity of AEs was reported as mild, moderate, and severe in 20.8%, 50.9%, and 26.4% of patients, respectively (Table 3). Severe-grade AEs reported by preferred term, irrespective of study drug relationship, included increased UPCR (3.8%), splenomegaly, atrial fibrillation, cardiac failure, goiter, cholestasis, hepatic failure, gastroenteritis, lower respiratory tract infection, lymph gland infection, urosepsis, femur fracture, spinal fracture, lumbar vertebral fracture, rib fracture, ulna fracture, transfusion reaction, arthralgia, back pain, malignant melanoma, papillary thyroid cancer, headache, device failure, renal colic, calculus urinary, hydronephrosis, and ureterolithiasis (1.9% each). None of the patients had severe GI AEs. Moderate- and severe-grade AEs by maximum severity grade were reported in 22/45 patients (48.9%) and 12/45 patients (26.7%) with deferasirox as prior chelation therapy, and 5/8 patients (62.5%) and 2/8 patients (25.0%) with other ICT as prior chelation therapy, respectively. Of the 53 patients, moderate-grade AEs were reported in 15 patients (28.3%) in the 7 to < 21 mg/kg/day daily-dose group and 12 patients (22.6%) in the ≥ 21 mg/kg/day daily-dose group, while severe-grade AEs were reported in 7 patients each (13.2%) in the 7 to < 21 mg/kg/day and ≥ 21 mg/kg/day daily-dose groups, respectively.

Serious AEs (SAEs) regardless of study drug relationship were reported in 13 patients (24.5%; MDS, n = 3; TDT, n = 10) and none of these were considered treatment related. SAEs, reported in 3 patients with MDS, included cardiac failure, device failure, femur fracture, cholestasis, hepatic failure, and malignant melanoma reported in 1 patient who died; lumbar vertebral fracture (in 1 patient); and urosepsis (in 1 patient). Other SAEs reported in patients with TDT included spontaneous abortion, atrial fibrillation, biliary colic, urinary calculus, cholecystitis, diverticulitis, fracture, goiter, hydronephrosis, lower respiratory tract infection, lymph gland infection, ovarian adenoma, panic attack, papillary thyroid cancer, renal colic, rib fracture, ulna fracture, and ureterolithiasis. None of these SAEs were reported in more than 1 patient (1.9% each). Death not suspected to be treatment related occurred in a 72-year-old male patient (1.9%) with very low–risk MDS (as per the Revised International Prognostic Scoring System [IPSS-R]). The cause of this on-treatment death according to clinical judgment was malignant melanoma with multiple metastasis in the liver and spleen, with unknown primary origin. Another contributing factor for the death was liver failure and intrahepatic cholestasis.

AEs leading to discontinuation of study treatment were reported in 4 patients (7.5%), of which drug ineffective AE in 1 patient (1.9%) and serum ferritin abnormal AE in 1 patient (1.9%) were considered treatment related. Treatment was discontinued due to an AE (moderate in severity, not suspected to be treatment related) in 1 MDS patient. AEs that led to dose adjustment/interruption occurred in 33 patients (62.3%), with the most frequently reported (> 10%) being increased UPCR (8 patients [15.1%]) and vomiting (6 patients [11.3%]). Treatment-related AEs that led to dose adjustment/temporary interruption were reported in 15 patients (28.3%) and included increased UPCR (6 patients [11.3%]), increased blood creatinine (3 patients [5.7%]), gastritis, glycosuria, proteinuria, upper abdominal pain (2 patients [3.8%] each), and diarrhea, gastric ulcer, increased serum ferritin, hypertransaminasemia, and increased transaminases (1 patient [1.9] each). AEs required additional treatment in 4 patients with TDT (7.5%; macular edema and skin ulcer in 1 patient [1.9%], diarrhea, radius fracture, and breast discomfort in 1 patient [1.9%] each), and none were suspected to be treatment related.

#### Adverse events of special interest

Overall, 22 patients (41.5%) reported AEs of special interest, of which AEs suspected to be treatment related were reported in 11 patients (20.8%). Common AEs of special interest (> 5% incidence) included increased UPCR (8 patients [15.1%]; severe and suspected to be treatment related: 2 patients [3.8%]), proteinuria (4 patients [7.5%]), increased blood creatinine (4 patients [7.5%]; MDS, n = 1), and hypertransaminasemia (4 patients [7.5%]). Hepatic failure due to metastatic liver disease (MDS, n = 1) and transfusion reaction (TDT, n = 1) of severe grade, but not suspected to be treatment related were reported in 1 patient each. One patient with MDS discontinued the treatment due to decreased creatinine renal clearance (moderate in severity, but not suspected to be treatment related) (Additional file 1: Table S3).

#### Laboratory parameters

Worst post-baseline elevations in SCr of > ULN at 2 consecutive measurements at least 7 days apart occurred in 2 patients (3.8%; MDS, n = 1; TDT, n = 1). One patient with MDS had worst post-baseline UPCR > 113.1 mg/mmol at 2 consecutive measurements at least 7 days apart (notable range). Two patients (3.8%) with MDS had worst post-baseline CrCl value within the notable range (< 60 mL/min at 2 consecutive measurements at least 7 days apart), and 2 patients (3.8%) had worst post-baseline CrCl < 40 mL/min (1 value). Two patients (3.8%) with TDT had a worst post-baseline ALT level in the notable range (> 5 × ULN and 2 × baseline value). Elevations of transaminases (AST or ALT) > 10 × ULN were uncommon (1.9%); only 1 patient (1.9%) with TDT had a post-baseline increase in AST and ALT values > 10 × ULN and > 2 × baseline value (Table 4).

**Table 4:** Shift tables of laboratory values based on notable/extended ranges

	Baseline	Worst post-baseline value				
ALT (U/L)	n (%)	≤ ULN, n (%)	> ULN to ≤ 5 × ULN, n (%)	> 5 × ULN, n (%)	Notable range, n (%)	Extended range, n (%)
≤ ULN	34 (64.2)	19 (55.9)	14 (41.2)	0	0	1 (2.9)
> ULN to ≤ 5 × ULN	19 (35.8)	0	17 (89.5)	0	2 (10.5)	0
Total	53 (100.0)	19 (35.8)	31 (58.5)	0	2 (3.8)	1 (1 .9)

AST (U/L)	n (%)	≤ ULN, n (%)	> ULN to ≤ 5 × ULN, n (%)	> 5 × ULN, n (%)	Notable range, n (%)	Extended range, n (%)
≤ ULN	41 (77.4)	23 (56.1)	17 (41.5)	0	0	1 (2.4)
> ULN to ≤ 5 × ULN	12 (22.6)	0	12 (100.0)	0	0	0
Total	53 (100.0)	23 (43.4)	29 (54.7)	0	0	1 (1.9)

Serum creatinine	n (%)	≤ ULN, n (%)	Two consecutive > ULN, n (%)	Notable range, n (%)	Missing, n (%)	
≤ ULN	53 (100.0)	51 (96.2)	2 (3.8)	0	0	
Total	53 (100.0)	51 (96.2)	2 (3.8)	0	0	

Creatinine clearance	n (%)	≥ 60, n (%)	One value ≥ 40 to < 60, n (%)	Notable range, n (%)	One value < 40, n (%)	Extended range, n (%)
≥ 60	50 (94.3)	45 (90.0)	2 (4.0)	1 (2.0)	2 (4.0)	0
≥ 40 to < 60	2 (3.8)	0	1 (50.0)	1 (50.0)	0	0
Missing	1 (1.9)	1 (100.0)	0	0	0	0
Total	53 (100.0)	46 (86.8)	3 (5.7)	2 (3.8)	2 (3.8)	0

Urinary protein/urinary creatinine ratio (mg/mmol)	n (%)	≤ 113.1, n (%)	One value > 113.1, n (%)	Two values > 113.1, n (%)	Notable range, n (%)	Missing, n (%)
≤ 113.1	48 (90.6)	39 (81.3)	5 (10.4)	3 (6.3)	1 (2.1)	0
> 113.1	1 (1.9)	1 (100.0)	0	0	0	0
Missing	4 (7.5)	3 (75.0)	0	0	0	1 (25.0)
Total	53 (100.0)	43 (81.1)	5 (9.4)	3 (5.7)	1 (1.9)	1 (1.9)

*ALT* alanine aminotransferase, *AST* aspartate aminotransferase, *ULN* upper limit of normal

#### Hematological parameters

The majority of patients had low RBC (41 of 53, 77.4%), hematocrit (49 of 53, 92.5%), and hemoglobin (49 of 53, 92.5%) values at baseline. Among 52 patients with a baseline platelet count ≥ 100 × 10^9^/L, only 2 patients (3.8%) had worst post-baseline values in the notable range (≥ 50 to < 100 × 10^9^/L). Similarly, among 52 patients with a baseline absolute neutrophil count ≥ 1.5 × 10^9^/L, the worst post-baseline values were found to be in the notable range (≥ 0.5 to < 1.5 × 10^9^/L) in 1 patient (1.9%) and extended range (< 0.5 × 10^9^/L) in 1 patient (1.9%). Mean ± SD change (mean relative percentage change) from baseline to month 6 and month 12 in key hematological parameters is represented in Additional file 1: Table S4.

## Discussion

This 2-year, phase 3, interventional study evaluated the efficacy and safety of deferasirox FCT in adult and pediatric patients (mean age, 32.9 years) with MDS (n = 4) and TDT (n = 49) who had previously completed 24 weeks of deferasirox treatment in the ECLIPSE study over a mean duration of further 571.5 days. Almost 80% of patients received an average actual deferasirox FCT dose of ≥ 17.5 mg/kg/day during the study. The average actual dose in MDS patients was comparatively less than that in TDT patients reflecting the notion of physicians using lower doses to treat MDS patients [2]. None of the patients were administered with higher than the maximum recommended doses of deferasirox FCT (28 mg/kg/day) [12].

Various studies have demonstrated that compliance with ICT significantly improves morbidity and mortality [4–6, 13]. Compliance (~ 90% mean relative consumed tablet count) and persistence (95–100%) rates with deferasirox FCT during this study were found to be on the higher side. Persistence rates (proportion of patients with continuous use of deferasirox FCT with no interruption for ≥ 30 days or ≥ 60 days) in this study are comparable to the real-world data on persistence in patients who switched from deferasirox DT (39–80%) to deferasirox FCT (60–96%) [1].

Common AEs (> 20%) reported with deferasirox FCT in this study (headache, diarrhea, pyrexia, nausea, vomiting, cough, and upper abdominal pain) were consistent with the known profile of deferasirox [2, 12]. AEs reported in ~ 70% of the patients were of mild to moderate in severity; none of the GI AEs were of severe grade nor led to discontinuation of treatment. These results were in line with those of previous studies, which reported that GI tolerability profile may be improved with the FCT compared with the DT. Lack of excipients that cause GI irritation and ease of administration along with a light meal could have contributed to the better GI tolerability of FCT [11, 13]. It is noted that the concomitant use of PPIs during the study was higher (32.1%) than reported prior use (18.3%). This study was not designed to investigate the reasons behind the increased use of PPIs, but we assume that they might have been prescribed as a general prophylaxis to avoid GI complications from other concomitant therapies (analgesics, antibiotics, etc.,) prescribed in comorbid conditions, infections, or procedures.

SAEs regardless of study drug relationship were reported in ~ 25% of patients and none (including the death due to malignant melanoma with multiple metastasis in the liver and spleen) were considered treatment related. Notably, of ~ 26 SAEs reported in 13 patients, 8 were reported in 3 MDS patients (aged ≥ 60 years) reflecting the considerable burden of the underlying hematological disorder together with the comorbidities of the affected elderly patients [14–16]. Discontinuation of treatment due to treatment-related AEs (drug ineffective and abnormal serum ferritin) during the 2 years was observed in only 2 TDT patients (3.8%). The low rate of SAEs and absence of treatment-related deaths in this study could have been a result of the greater compliance (~ 90%) and persistence (95–100%) rates with deferasirox FCT.

The ECLIPSE study reported that abnormal renal parameters/renal AEs were more common in patients receiving deferasirox FCT than in those receiving deferasirox DT due to an intake of higher than the recommended dose [11]. Most of the renal abnormalities/AEs reported during this 2-year study were mild and reversible with dosage adjustment or temporary interruption of deferasirox FCT. Only 1 patient discontinued the treatment due to decreased creatinine renal clearance, which was moderate in severity and not suspected to be treatment related. Increases in SCr and liver function tests in the notable/extended ranges were observed in some patients, and those increases were consistent with the known safety profile of deferasirox FCT [12]. No substantial difference in severity of all AEs was noted based on dosing groups (7 to < 21 mg/kg/day and ≥ 21 mg/kg/day) as well. Administration of the recommended doses of deferasirox FCT with constant dosage adjustment as per serum ferritin levels might have resulted in fewer renal and liver abnormalities in this follow-up study. These results highlight that the optimal benefit of iron chelation with minimal toxicity of deferasirox FCT can be achieved when administered at recommended doses with close monitoring for AEs and individualized dosage adjustment as per serum ferritin levels.

With respect to efficacy, a consistent reduction in serum ferritin level was observed across all post-baseline time points and > 25% decrease in mean relative change of serum ferritin  was observed from month 9 onward. The mean decrease (relative change in percentage from baseline) in serum ferritin in the current study was higher at month 12 than at month 6 (29% vs 19%), which was also greater than that observed with FCT (14%) and DT (4%) at 6 months in the ECLIPSE study [11]. Greater compliance (~ 90%) and persistence (95-100%) rates could have contributed to the observed serum ferritin reduction at month 12 in this study.

This study presents only descriptive statistics. The study results may not be generalized for treatment of MDS, as there were only 4 patients with MDS. This study was not designed to study any correlation of transfusion burden with iron overload; hence, no comment has been made on the impact of ferritin reduction on transfusion burden.

## Conclusions

Safety evaluation results of deferasirox FCT from this 2-year interventional study in adult and pediatric patients with chronic iron overload in TDT or MDS were consistent with the known safety profile of deferasirox. No new safety signals were identified and no major concerns associated with GI tolerability or renal safety were reported during the 2 years. Patients receiving FCT had a treatment compliance of ~ 90% and persistence of > 95%, with a consistent reduction in serum ferritin level starting from week 2 across all post-baseline time points. These results suggest that the recommended dosing of deferasirox FCT with better tolerability, palatability, and compliance, offers a favorable option of ICT for long-term management of iron overload and associated complications in TDT.

## Supplementary information

**Additional file 1: Table S1.** Summary of blood transfusions during the study. **Table S2.** Overview of adverse events. **Table S3.** Adverse events of special interest by preferred term (safety set). **Table S4.** Change from baseline to month 6 and month 12 in key hematological parameters.

## Data Availability

Key data generated or analyzed during this study are included in this published article and its additional files. Any additional datasets generated during and/or analyzed during the current study available from the corresponding author on reasonable request.

## Abbreviations

AE: Adverse event
ALT: Alanine aminotransferase
AST: Aspartate aminotransferase
CI: Confidence interval
CrCl: Creatinine clearance
DT: Dispersible tablet
FCT: Film-coated tablet
GI: Gastrointestinal
ICT: Iron chelation therapy
MDS: Myelodysplastic syndrome
RBC: Red blood cell
SAE: Serious adverse event
SCr: Serum creatinine
SD: Standard deviation
TDT: Transfusion-dependent thalassemia
UPCR: Urine protein to creatinine ratio
ULN: Upper limit of normal
