# Supplementary material for: Two-year long safety and efficacy of deferasirox film-coated tablets in patients with thalassemia or lower/intermediate risk MDS: phase 3 results from a subset of patients previously treated with deferasirox in the ECLIPSE study
Source: Exp Hematol Oncol. 2020 Aug 10;9:20. doi: 10.1186/s40164-020-00174-2 (PMC7419189; doi:10.1186/s40164-020-00174-2)
Supplement: Supplementary file 1 — Additional file 1: Table S1. Summary of blood transfusions during the study. Table S2. Overview of adverse events. Table S3. Adverse events of special interest by preferred term (safety set). Table S4. Change from baseline to month 6 and month 12 in key hematological parameters. [file 40164_2020_174_MOESM1_ESM.doc]

**Table S1 Summary of blood transfusions during the study**

| **Number of transfusions^a^** | **Deferasirox FCT**  **N=53**  **n (%)** |
| --- | --- |
| 1 to <10 | 4 (7.6) |
| 10 to <20 | 7 (13.2) |
| 20 to <30 | 7 (13.2) |
| 30 to <40 | 10 (18.9) |
| 40 to <50 | 14 (26.4) |
| 50 to <60 | 6 (11.3) |
| 60 to <70 | 3 (5.7) |
| 70 to <80 | 0 |
| 80 to <90 | 1 (1.9) |
| ≥90 | 1 (1.9) |

Abbreviation: FCT, film-coated tablet.

^a^The number of transfusions corresponds to the number of transfusion sessions, during which 1-3 units of packed red blood cells were given.

**Table S2 Overview of adverse events**

| **Category of AEs (N=53)** | **All AEs**  **n (%)** | **Treatment-related AEs**  **n (%)** |
| --- | --- | --- |
| Overall AEs | 52 (98.1) | 20 (37.7) |
| SAEs | 13 (24.5) | 0 |
| Fatal AEs | 1 (1.9) | 0 |
| AEs leading to discontinuation | 4 (7.5) | 2 (3.8) |
| AEs leading to dose adjustment/interruption | 33 (62.3) | 15 (28.3) |
| AEs requiring additional therapy | 4 (7.5) | 0 |

Abbreviations: AE, adverse event; SAE, serious AE.

**Table S3** Adverse events of special interest by preferred term (safety set)

| **Grouping**  **Preferred term** | **Deferasirox FCT**  **N=53**  **n (%)** | **SAE^a^** | **AE suspected to be treatment related^b^** | **Discontinued from study owing to AE** |
| --- | --- | --- | --- | --- |
| Number of subjects with at least one event | **22 (41.5)** | **4 (7.5)** | **11 (20.8)** | **1 (1.9)** |
| Gastrointestinal hemorrhages ulcers esophagitis | **1 (1.9)** | **0** | **1 (1.9)** | **0** |
| Gastric ulcer | 1 (1.9) | 0 | 1 (1.9) | 0 |
| Hearing loss | **2 (3.8)** | **0** | **0** | **0** |
| Hypoacusis | 1 (1.9) | 0 | 0 | 0 |
| Neurosensory hypoacusis | 1 (1.9) | 0 | 0 | 0 |
| Increased liver transaminases, hepatic failure, hepatitis (excluding infections) | **7 (13.2)** | **1 (1.9)** | **2 (3.8)** | **0** |
| Hypertransaminasemia | 4 (7.5) | 0 | 1 (1.9) | 0 |
| Transaminases increased | 1 (1.9) | 0 | 1 (1.9) | 0 |
| Hepatic failure | 1 (1.9) | 1 (1.9) | 0 | 0 |
| Alanine aminotransferase increased | 1 (1.9) | 0 | 0 | 0 |
| Lens opacities, retinal changes, optic neuritis | **2 (3.8)** | **0** | **0** | **0** |
| Vision blurred | 1 (1.9) | 0 | 0 | 0 |
| Macular edema | 1 (1.9) | 0 | 0 | 0 |
| Peripheral blood cytopenias | **3 (5.7)** | **1 (1.9)** | **0** | **0** |
| Transfusion reaction | 2 (3.8) | 1 (1.9) | 0 | 0 |
| Neutropenia | 1 (1.9) | 0 | 0 | 0 |
| Renal disorders (increased serum creatinine, acute renal failure, renal tubular disorder, acquired Fanconi's syndrome) | **15 (28.3)** | **2 (3.8)** | **11 (20.8)** | **1 (1.9)** |
| Increased urine protein/creatinine ratio | 8 (15.1) | 2 (3.8) | 6 (11.3) | 0 |
| Proteinuria | 4 (7.5) | 0 | 3 (5.6) | 0 |
| Increased blood creatinine | 4 (7.5) | 0 | 4 (7.5) | 0 |
| Decreased creatinine renal clearance | 1 (1.9) | 0 | 0 | 1 (1.9) |

Abbreviations: AE, adverse event; FCT, film-coated tablet; SAE, serious adverse event.

^a^Higher grade of severity was considered if various severities were reported for an AE in a single patient; ^b^AE of a preferred term was reported only once for one patient.

**Table S4 Change from baseline to Month 6 and Month 12 in key hematological parameters**

| **Hematological parameter** | **Baseline** | **Month 6** | **Month 12** |
| --- | --- | --- | --- |
| RBC count (× 10^12^/L),  mean (SD) | **n=53** | **n=42** | **n=40** |
| Baseline | 3.8 (0.49) | 3.7 (0.50) | 3.7 (0.51) |
| Change from baseline |  | −0.1 (0.39) | −0.1 (0.43) |
| Relative change from baseline |  | −2.9 (10.34) | −3.3 (11.74) |
| WBC total (× 10^9^/L),  mean (SD) | **n=53** | **n=42** | **n=40** |
| Baseline | 9.3 (5.14) | 9.6 (5.67) | 9.1 (5.47) |
| Change from baseline |  | −0.5 (4.07) | −0.1 (4.58) |
| Relative change from baseline |  | 3.8 (33.43) | 9.5 (47.22) |
| Platelet count (× 10^9^/L),  mean (SD) | **n=53** | **n=42** | **n=40** |
| Baseline | 330.1 (188.81) | 336.5 (195.97) | 339.4 (196.41) |
| Change from baseline |  | 7.7 (112.98) | 22.4 (100.72) |
| Relative change from baseline |  | 10.3 (40.78) | 14.9 (27.98) |
| Hb (g/L),  mean (SD) | **n=53** | **n=42** | **n=40** |
| Baseline | 103.6 (11.95) | 103.1 (11.62) | 102.5 (12.12) |
| Change from baseline |  | −4.5 (10.76) | −3.5 (13.23) |
| Relative change from baseline |  | −4.0 (10.52) | −2.7 (12.86) |
| Hematocrit (L),  mean (SD) | **n=53** | **n=42** | **n=40** |
| Baseline | 0.3 (0.03) | 0.3 (0.03) | 0.3 (0.03) |
| Change from baseline |  | −0.0 (0.03) | −0.0 (0.04) |
| Relative change from baseline |  | −2.6 (10.19) | −2.8 (13.60) |

Abbreviations: Hb, hemoglobin; SD, standard deviation; RBC, red blood cell; WBC, white blood cell.
